# Learned modesty and the first lady's comet: a commentary on Caroline Herschel (1787) ‘An account of a new comet’

**DOI:** 10.1098/rsta.2014.0210

**Published:** 2015-04-13

**Authors:** Emily Winterburn

**Affiliations:** Department of Philosophy, Religion and History of Science, University of Leeds, Leeds, UK

**Keywords:** Caroline Herschel, comets, women in science, astronomy, eighteenth century

## Abstract

Long before women were allowed to become Fellows of the Royal Society, or obtain university degrees, one woman managed to get her voice heard, her discovery verified and her achievement celebrated. That woman was Caroline Herschel, who, as this paper will discuss, managed to find ways to fit comet discoveries into her domestic life, and present them in ways that were socially acceptable. Caroline lived in a time when strict rules dictated how women (and men) should behave and present themselves and their work. Caroline understood these rules, and used them carefully as she announced each discovery, starting with this comet which she found in 1786. Caroline discovered her comets at a time when astronomers were mainly concerned with position, identifying where things were and how they were moving. Since her discoveries, research has moved on, as astronomers, using techniques from other fields, and most recently sending experiments into space, have learned more about what comets are and what they can tell us about our solar system. Caroline's paper marks one small, early step in this much bigger journey to understand comets. This commentary was written to celebrate the 350th anniversary of the journal *Philosophical Transactions of the Royal Society*.

In consequence of the Friendship which I know to exist between you and my Brother I venture to trouble you in his absence with the following imperfect account of a comet [1].

This is how Caroline Herschel began her letter to Charles Blagden, then secretary of the Royal Society, announcing, and claiming priority for, her first comet. The comet is known to astronomers today as Comet C/1786 P1 (Herschel) and was found, she tells us in this paper, on 1 August 1786 between the constellations Ursa Major and Coma Berenices. The paper is beautiful in its simplicity. In it she states concisely and succinctly the circumstances of her discovery, her reasons for concluding that it was a comet and her evidence for that claim. She then describes where the comet was found and how its position changed night by night, enabling others to verify her claim and calculate its orbit. The paper ends with a short description of her apparatus including her telescope's focal length and magnifying power.

Her paper includes sketches and accompanying descriptions of the comet's position at different times in relation to three stars labelled *a*, *b* and *c*. Using her knowledge of geometry, which she has described elsewhere as having learned from her brother William Herschel, she depicts the relationship between the stars and the comet in terms of triangles. On 1 August at 10 h 33' she drew the comet explaining that it makes a ‘perfect isoseeles [sic] triangle with the two stars *a* and *b*.’ Later she portrayed the comet as making ‘an obtuse triangle’ with two numbered stars in Ursa Major and three stars in Coma Berenices. Unhelpfully, she states that she could not find stars *a*, *b* or *c* in any catalogue (figure 1).

**Figure 1.**
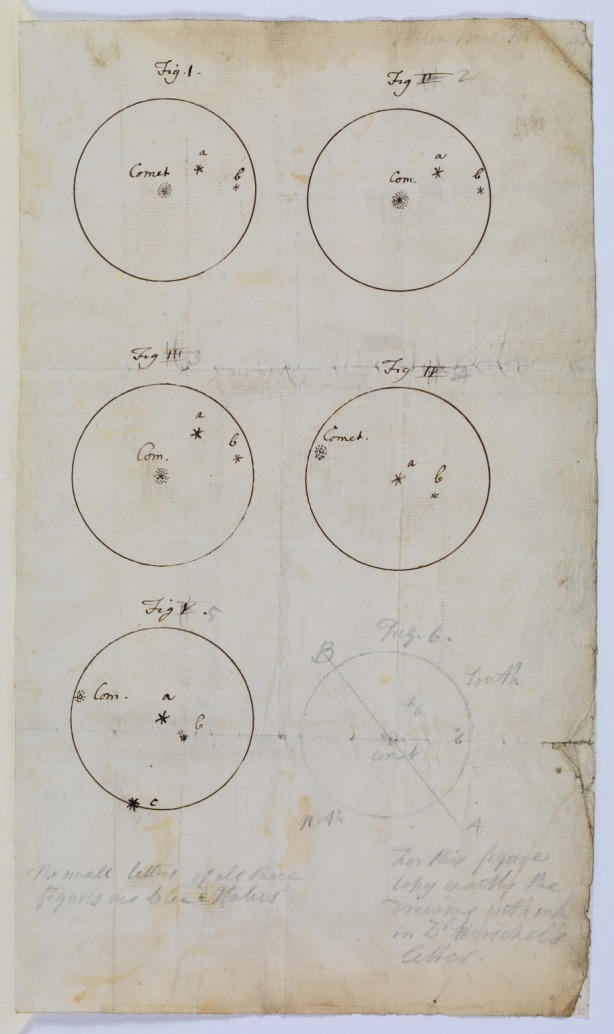
Her page of sketches accompanying her letter showing the comet against various combinations of stars ‘*a*, *b* and *c*’. Copyright The Royal Society.

In a footnote, written a few days after the original letter, her brother William added that he too had looked in the region of the sky Caroline described to try to identify stars *a* and *b*. He found, however, ‘so many small stars in that neighbourhood that I have not been able to fix on any of them that will exactly answer these figures’. He even suggested, given the light (moonlight, twilight, the stars being near the horizon) that she may have been mistaken in these observations though he quickly followed this with an explanation of why this should not invalidate her claim to the comet's discovery.

The language Caroline used in this paper highlights her awareness of both the proper codes of conduct for women, and the expectations of the scientific community. On the one hand, her language is modest, self-effacing and polite. She ‘venture[s] to trouble’ the recipient, and only does so because of ‘the friendship [that] I know exists between you and my brother’. She explained her brother's absence, and hence why she was observing and why it was she and not her brother who was writing. She then ended her paper not with any overt claims to priority, but rather to ask that he ‘will do me the favour of communicating these observations to my brother's astronomical friends.’ This contrasts starkly with the language her brother used in his paper announcing his discovery of a comet (which later turned out to be the planet Uranus). Like Caroline's, the paper was in the form of a letter. In William's letter, however, there is no dressing up the announcement as an interesting piece of information to share with friends and friends of the family. Rather this is quite clearly and unapologetically a claim for priority. The paper begins with a description of William's observations concluding ‘[t]he sequel has shewn that my surmises [sic] were well founded, this proving to be the Comet we have lately observed’ [2].

Caroline clouded her announcement in social, almost domestic remarks about her brother's friends. At the same time, she astutely identified the information required to make hers a genuine scientific claim for priority. She explained the origin of her abilities, that most of her time was usually spent in the ‘employment of writing down the observations, when my Brother uses the 20 feet reflector’. She explained William's absence ‘he is now on a visit to Germany’, and went on to detail the circumstances that led to her discovery. ‘I have taken the opportunity of his absence to sweep in the neighbourhood of the Sun, in search of Comets’. For each described observation she then gave exact times and where possible, locations in terms of named stars. The paper ends with a description of her apparatus, her methods and why she drew her conclusions. The paper is only two handwritten pages long, three including the diagrams, four when William's footnote is added. Yet she managed to pack in both her self-effacing, yet authoritative cushioning, and a succinct and rigorous account of her observations, conclusions and defence of their credibility. It is an impressive achievement, and delicately done.

Despite the neatness of its construction, this 1787 paper derives much of its importance not from its content so much as from its author. This was the first paper by a woman to be read to the Royal Society. It was one of the first papers by a female author to appear in any scientific journal throughout the world. It did not open the doors to female participation in such learned societies—the Royal Society did not elect its first female fellow until 1945—but it did introduce the prospect that such a change might one day be possible.

Caroline Herschel's story, generally told alongside that of her brother William's, is well known in the history of science. The Herschels grew up in a musical family in Hanover, and there Caroline was trained to look after her brothers and ageing parents. She joined her brother, William in England in 1772, ostensibly to train as a singer and to accompany him in his concerts. This training was technical in nature, teaching her the mechanics of how to sing and make her voice carry, how to read music and speak English. She was also taught mathematics, so that she might take over management of the household accounts. These began as ‘little lessons for Lina’, but soon developed into more abstract teachings: ‘A little Geometry for Lina’, ‘A little Algebra for Lina’ and so on.^1^

In addition to these musical, linguistic and mathematical lessons, Caroline's musical education while in Bath also involved lessons in how to perform, and crucially how to present herself in concerts and socially among their musical clients. It was through these lessons that Caroline learned the appropriate ways in which a woman must present herself and her work. It was a lesson she learned most memorably through an exchange with an unnamed woman in Bath who told her off for ‘being my own Trumpeter’ [3]. By applying these lessons of female modesty, self-depreciation and politeness, Caroline was later able to couch her astronomical work in ways that allowed it to be welcomed and accepted.

Gradually, Caroline became more and more embroiled in her brother's work. Then in 1781 William discovered the planet Uranus and this gained him a Royal Pension from George III, with the requirement that they should give up music and move near to Windsor Castle. Once there, Caroline began training as an astronomer, learning skills she was expected to put to use almost immediately, acting as her brother's astronomical assistant and scribe. She began to learn more mathematics too, quizzing her brother whenever she got the chance. As she told her nephew, these lesson chiefly took the form of ‘answers of your fathers to the inquiries I used to make when at breakfast, before we separated, each for our dayly tasks, &c. &c.’^2^ As she had throughout her life, Caroline used any spare time she had free from assisting others, to pursue tutoring and then to practice alone. It was during one of these solitary practice sessions, alone with her telescope, ‘sweeping’ as she put it the night sky, gradually improving her knowledge of it and her mastery of observing, that she discovered her first comet.

The paper that followed was the first in a series announcing in all eight comets discovered by Caroline. This first discovery followed roughly 5 years of observing practice, and several years of assisting William in his observations, and writing out his scientific papers for him. Through helping William she had learned what was required in a scientific paper. She had also become familiar enough with the night sky, for the appearance of a comet to seem noteworthy and out of the ordinary.

Caroline wrote the paper while her brother was away. Interestingly, all eight of her comet discoveries were made while she lived in her brother's home, yet on days when he was otherwise occupied [4]. In each case, she ensured that her discovery was announced as quickly as possible, and to an influential friend. In the case of her eighth comet, she even took the precaution of riding her horse through the night, to Greenwich to announce her discovery to the Astronomer Royal, Nevil Maskelyne rather than risk losing priority. These actions rather undermine her self-depreciating claims of wishing only, as she put it in one paper, to have her comets announced ‘for the sake of astronomy.’ [5].

At the time of this first discovery several hundred comets had already been identified. This one, however, was regarded as special and important because it was the first ‘lady's comet’. Fanny Burney, the novelist, diarist and lady-in-waiting to Queen Charlotte reported viewing this comet on a visit to the Royal Palace. ‘The comet’ she wrote ‘was very small, and had nothing grand or striking in its appearance; but it is the first lady's comet, and I was very desirous to see it.’^3^ Similarly, in 1787 Sir Joseph Bank, then President of the Royal Society, suggested Caroline's Royal pension should come from Queen Charlotte rather than George III, since Caroline was ‘the lady's comet hunter’ [6] (figure 2).

**Figure 2.**
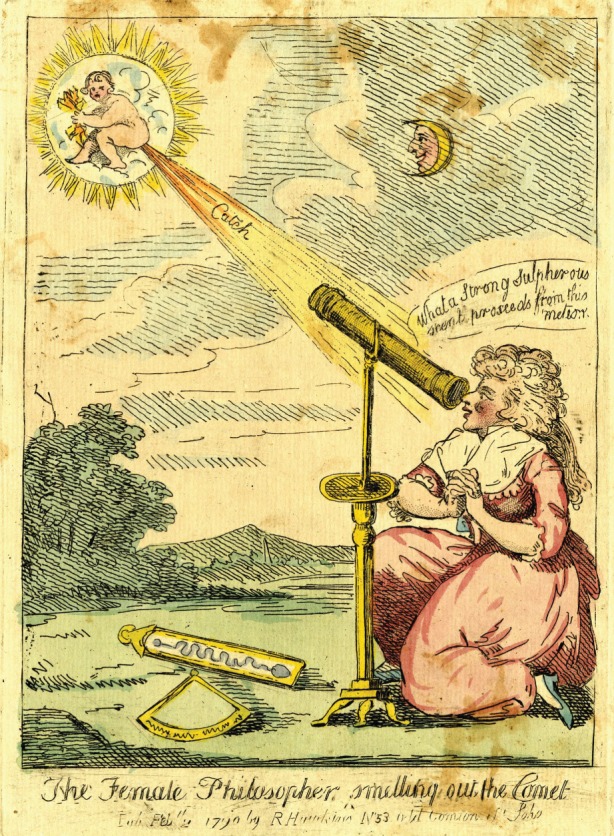
The Female Philosopher: Smelling out the Comet, 1790s. (Draper Hill Collection, The Ohio State University Billy Ireland Cartoon Library & Museum.)

The attention Caroline attracted as the first female comet hunter was not always flattering or complimentary. Though it's hard to say exactly what the cartoonist here is ridiculing, the image implies that a woman seeking comets was regarded by some as a figure of fun. Many members of the scientific elite, however, were more generous, and keen to celebrate this woman in a male-dominated field. Astronomer, mathematician and friend of William Herschel, Jérôme de Lalande made a point of emphasizing Caroline's importance as a lady astronomer, introducing her whenever possible to all and any female philosophers and mathematicians that crossed his path. One lady in particular was mentioned several times in Lalande's correspondence with the Herschels. Madame Louise du Piery was a student of Lalande in 1779 and went on to become the first female professor at the Sorbonne in 1789. The correspondence, in which Lalande encouraged both women to admire the others separate but complementary achievements and abilities, can be read as a lesson in self-presentation. Each emphasized what the other could do, insisting they were envious of their prowess and inspired to study harder. Neither could be accused of ‘being their own Trumpeter’, allowing Lalande to generously perform that task for them himself.^4^

Caroline's paper is an announcement of the existence of a previously unknown comet, so simply within those terms it is as factually accurate today as it was when it was written. The comet exists and had not been spotted before (unlike some of Caroline's later comet discoveries). Her paper makes no attempt to analyse the comet's orbit, or make predictions about its future position or date of return. It does not consider the composition of the comet or where it originated. While our knowledge of all these areas of comet study are much more developed now than when Caroline wrote her paper, they go beyond what would reasonably have been expected at the time.

The field of comet studies has gone through several distinct phases since Caroline's discovery. The eighteenth century, in which all eight of Caroline's comets were discovered, was a period dominated by positional astronomy. Comets were identified and observed, their positions noted and their orbits calculated. In 1705, Edmund Halley successfully predicted the return of the comet that now bears his name using Isaac Newton's new laws and historical records of comet observations. As he suggested, the comet returned in 1758, showing that comets were part of the solar system and had predictable orbits. Caroline's seventh comet, found by her in 1795, and previously sighted by French comet hunter Pierre Méchain in 1786, was the second comet to have its orbit successfully calculated. This comet, 2P/Encke was named after the person who made those calculations, the German astronomer Johann Franz Encke.

Increasingly refined observations and calculations of comets and their orbits followed and Caroline's first comet was one of those studied. Pierre Méchain, and much later (in 1918) Margaretta Palmer, both attempted to calculate possible orbits for the comet [7]. Both came up with parabolic orbits, Palmer also calculated four elliptical orbits with a broad range of possible periods, all several thousand years long. The variation Palmer put down to the accuracy of measurements made at the time.

Margaretta Palmer was a student of Maria Mitchell, the American astronomer who in 1847 became the second woman to discover a comet. Palmer had based her doctoral thesis on the recalculation of the orbit of Maria Mitchell's 1847 comet. Her work on Caroline's comets represents some of her post-doctoral research. In the introduction to her doctoral thesis Palmer remarked that her choice of which comets to study came in part from their having been discovered by female astronomers. In that one small claim, we begin to get a sense of the impact of Caroline's paper on if not the study of comets itself, then certainly on the women entering that field.

Around 1860 cometary investigations entered a new phase. Still using mathematics and observational data, Giovanni Schiaparelli (better known for his ‘canals’ on Mars theories) worked out a relationship between comets and meteor showers. The great Leonid meteor shower of 1833 had sparked new interest in this field, and through careful study Schiapparelli was able to show meteor showers were the result of the Earth's orbit passing through the debris left by a comet. Specifically, he was able to link the Leonid meteor shower with the comet Tempel–Tuttle and the Perseid shower with the comet Swift–Tuttle.

Alongside Schiapparalli's discovery, which like Halley and Encke's had been worked out from historical records, came new approaches to cometary investigations. In the nineteenth century, astronomy began to make use of emerging techniques from physics and chemistry, most notably spectroscopy, to investigate the composition of celestial bodies. In the 1860s this technique was applied to the study of comets.

Giovanni Battista Donati, William and Margaret Huggins were all important figures in the application of spectroscopy to the study of comets. Donati, whose 1858 comet became the first comet to be photographed, was the first to apply spectroscopy to the study of comets. The comet was comet 1864b, and Huggins helped him interpret his findings. Investigations over the following years looked at the spectra of many more comets, also distinguishing between composition of the nucleus and the comet's coma and tail.

Increasingly, refined studies and calculations of comets’ orbits have shown that comets fall into two main orbital categories: short- and long-period comets. In the mid-twentieth century, two theories emerged as to the origins of these different types. In the 1940s and 1950s, Gerard Kuiper and Leonard Edgeworth postulated the existence of a belt beyond Neptune housing short-period (less than 200 years) comets. Around the same time, Jan Oort came up with a cloud even further out, reaching from the edge of the Kuiper belt all the way out and beyond, to account for comets of longer period orbits.

Research on the composition of comets also continued throughout the twentieth century. Gradually, a picture emerged suggesting comets were made up of dust and ice leading Fred Whipple to famously coin the term ‘dirty snowball’ as a description in 1964. Research in this century has begun to revise this model, suggesting that some comets at least display different ratios of materials and might better be described as icy dirtballs. To confuse matters further, some asteroids have been found to contain similar materials, blurring the lines between these two types of celestial body previously considered distinct.

One of the most significant developments in cometary research over the last 50 years has been through the use of space missions to study comets close up. In 1986, ESA's first deep space spacecraft came within 600 km of Halley's Comet, providing the Earth with its first clear view of a comet's nucleus. NASA's spacecraft Spacedust meanwhile was able to fly alongside Comet Wild 2 in 2004, close enough to collect samples which were sent back to Earth for analysis. More recently still, the Rosetta Mission is, as I write this (November 2014), depositing a lander on the surface of the nucleus of Comet 67/P Chuyumov–Gerasimenko to study composition of this part of the comet. The comet chosen for this investigation was that discovered by Svetlana Gerasimenko and her colleague Klim Ivanovich Chuyumov on an expedition to Alma-Ata in 1969.

In these, and other space missions, women, although still always in the minority, have played recognized roles within the project teams. On the Rosetta spacecraft, for example, two out of the 11 scientific experiments included have been led by women. One of these, Giada (the Grain Impact Analyser and Dust Accumulator), was led by Alessandra Rotundi from Naples. The other, Rosina (the comet pressure sensor) was led by Kathrin Altwegg of Bern.

Caroline and her comet discoveries form part of the first wave of this long history of comet research. Like a number of her contemporaries she was interested in sweeping the sky, searching out new comets to add to existing lists. Her discovery served as one small part of this steady accumulation of data that would in time yield our current understanding of what comets are and how they behave. It is the fact that the comet discussed in Caroline's paper was discovered by a woman that makes this discovery stand out among those of her contemporaries.

The history of women in science is a field that has developed considerably over the last century. With each new generation, there has been a subtly different understanding of Caroline Herschel's significance and the importance of this and her other comet discovery announcements. In terms of modern scholarship, Caroline's paper is informative in highlighting some of the rules governing female engagement within the scientific community in the late eighteenth century.

There is now a considerable body of work looking at and rediscovering women and the role they have played in science over the centuries. We now know that Caroline Herschel was one of many women involved in the scientific work of family members and friends in the eighteenth century. While she used to be regarded as unusual for her involvement, we now see that her uniqueness came instead from her ability to get that work recognized. While many women participated in science in the eighteenth century, in most cases their contributions were largely eclipsed by their male collaborators. Just as servants, technicians and instrument makers played a part in scientific work, but were rarely named or officially acknowledged, so too were many wives, sisters and daughters involved but often left uncredited. What makes Caroline unusual was that her contributions were publically acknowledged in her own time, and have since been remembered by historians.

Caroline's paper gives us an insight into how it was that she came to take on this unusual female role in science. We can see by her careful use of language, her use of friendships—her own and her brothers—and by her restrained claims to innovation that she was intensely aware of how her words might be taken. She was aware of the power of presentation, and, amazingly, was able to use that to her own advantage.

Caroline Herschel's reputation historically has been based on two things: her contributions to her brother's astronomical work, and her own discovery of comets. Early twentieth century histories of women in science tended to emphasis the former, including her in titles such as *Famous Sisters of Great Men*, *The Romance of a Woman*'*s Influence* and *I Had a Sister* [8–10]. In more recent years, this approach has produced some innovative studies of male–female couples in science, of which William and Caroline are a frequently featured example. These studies have allowed us to see patterns in the way households divided tasks [11–13]. It has also given us some insights into the barriers facing women, and how often a male champion such as a husband or brother could help those barriers to be managed, if not actually overcome. It is through this approach, to understanding science as a collaborative exercise with many historically uncredited participants, that many previously forgotten women have been recovered.

When it became popular towards the end of the twentieth century to find tangible contributions made by women in science so they might sit alongside the ‘great men’, Caroline and her comets became an obvious candidate [14–16]. Several problems with this approach to returning women to the story of science however have begun to surface in recent years, and, again, discussion of Caroline and her comets have played their part. One of the problems has been that women have, for a variety of educational, social and cultural reasons, historically taken on different roles in science to men. The values we place on these different roles often has social undercurrents, so, for example, tasks carried out by women, and other low status groups, have historically been labelled less ‘scientific’ than those carried out by white upper class men. To make women's roles fit into this model of science, exaggerated claims have sometimes been made of their contribution, while the barriers they faced have been down played. Some of these tensions can be seen played out in the differing interpretations of Caroline's contribution to science. While Michael Hoskin, for example, considered Caroline to be a ‘mere assistant’, Claire Brock, basing her assessment on the same evidence, preferred to characterize her as ‘a practicing astronomer in her own right’ [17,18].

In reviewing the literature on Caroline Herschel up to 2002, Patricia Fara warned against this tendency to exaggerate Caroline's independent scientific achievements. Instead she suggested that it was our understanding of what constitutes a proper scientific contribution that needed to change [19]. This debate is best understood within the context of a wider discussion. During the 1980s and 1990s feminist historians of science began to look at the cultural origins of research questions, terminology and analogies in science and what that meant for the production of new knowledge [20,21]. This has been seen most starkly in the analysis of biological models, but it began to show us that no question or choice could be regarded as entirely neutral.

At the same time, feminist, and indeed historians looking at other historically marginalized groups, started to look at how we define and give differing status to the various roles within the scientific process. In doing so, they have begun to look at what this has meant for recovering hidden participants such as women, servants and technicians.^5^ Work in this field continues today with studies on the ‘circles’ surrounding and helping to make possible the work of celebrated historical figures. Through this work, it has become apparent that our definition of what constitutes science has, historically, been heavily dependent on our notions of a social hierarchy.

Feminist histories of science have now moved on in many different directions. One strand that is perhaps most interesting and relevant to our interpretation of this paper by Caroline Herschel concerns our understanding of self-presentation within the scientific community. Here the strategies used by a number of European women have been studied, looking at how they went about negotiating a place for themselves in the scientific world. Caroline's paper fits very well into this narrative. Like these women, as we have seen in her introductory and closing remarks in particular, Caroline learned the rules of appropriate female etiquette and then applied them to the scientific world [22–26].^6^ Again, like earlier histories, this approach helps us to understand how the social and scientific worlds of the eighteenth century interacted. They may even help us understand how these social rules of language and self-presentation continue to sustain gender imbalance in science today.

Caroline Herschel's 1787 paper demonstrates her awareness and skilful use of these social rules of female modesty and demure respectability. Her awareness of these rules however can be found elsewhere too, in portraiture for example. Scientific portraits generally conform to certain conventions whereby the sitter is shown at work, or surrounded by the tools of their trade [27]. Caroline sat for only two portraits in her lifetime; neither could be described as traditional scientific portraits. Both portraits show Caroline demurely dressed, head covered, collar buttoned up to the chin with minimal clutter around her. In neither image are there any props or background imagery to indicate her importance or profession. One shows her leaning on a picture of the solar system, her finger resting on the orbit of a comet, but again, it is a subtle, understated representation of her work. Like her writing, these portraits show how careful Caroline, and other women in her position, needed to be in their self-presentation, and how very different the rules were for women than for their male contemporaries (figure 3).

**Figure 3.**
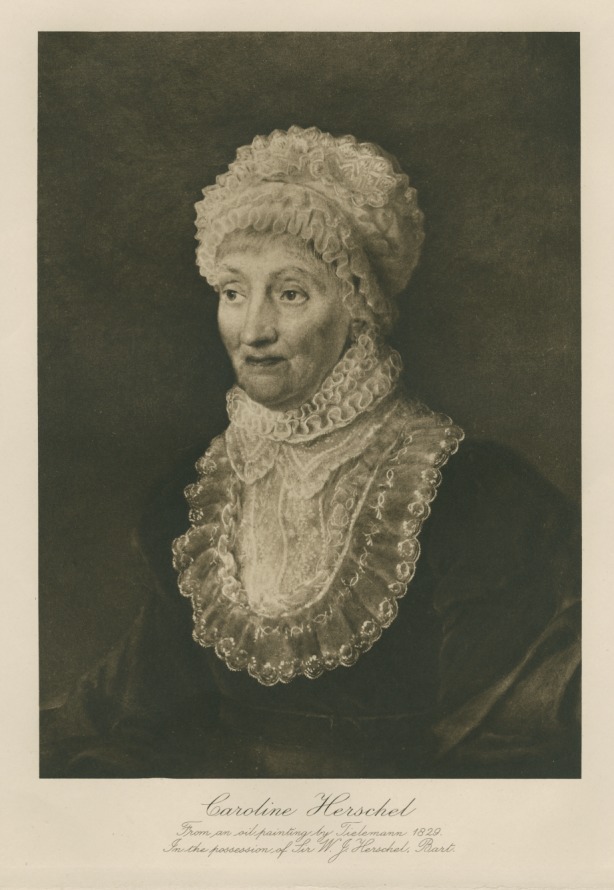
Caroline Herschel by M. G. Tieleman, 1829. Copyright The Royal Society.

While, as we have seen, Caroline's status as a female discoverer of comets helped inspire Maria Mitchell's student Margaretta Palmer, her influence was felt perhaps most strongly within her own family. Her nephew's wife, Margaret Brodie Herschel (née Stewart) was intensely interested in her famous astronomer aunt and quizzed her about her life to such an extent that Caroline declared Margaret to have been the impetus behind her decision to write her autobiography. She was remembered and admired by the next generation too. Margaret's daughter-in-law Mary Cornwallis Herschel was behind the publication of Caroline's memoirs in 1876, which helped recover Caroline from impending obscurity. Margaret's youngest, Constance, meanwhile a few decades later gave Caroline equal billing with her more famous brother William when she wrote what has become the definitive introduction to the Herschels, *The Herschel Chronicles* in 1933. Margaret's eldest daughter, named Caroline after her illustrious great aunt, gave Girton College (where her sister Constance studied) her great aunt's gold medal from the Royal Astronomical Society. The medal was to sit alongside Mary Somerville's library, so that they might ‘keep green among generations of students still unborn the memory of the two illustrious women who…showed the way to follow’ [28].^7^

The study of women in science is now a broad field, encompassing the study of consistently high profile women such as Caroline Herschel, as well as hidden figures. This secondary category often contributed in ways that have been previously ignored owing to the woman's status as wife, mother, sister, daughter or indeed simply because she was of a low and therefore invisible social class. Caroline's story, and the skills she displays in this paper, gives an insight not only into the behaviour and successes of the handful of high profile women in the history of science. Her example also gives us new tools in understanding those women who were less visible. Through her story we can see the choices available to an eighteenth century participant in science, and why some may have chosen not to draw attention to themselves, or indeed may have simply not known how.

## Footnotes

1 ‘Little Lessons for Lina, RAS: WH.7/13.

2 Set of papers titled ‘chiefly answers of your fathers….’ BL: microfilm M/588(5).

3 Fanny Burney's diary for the year 1786 as quoted in Lubbock [5, note 6, p. 169].

4 RAS WH13.L.3–6 & L.8

5 See, for example, the special issue on ‘Technicians’ of *Notes and Records of the Royal Society*, **62**, 1 (2008).

6 Also Emily Winterburn, ‘Caroline Herschel, agency and self-presentation’, *Notes and Records of the Royal Society*, March 2015.

7 Quote from ‘reprint of Girton Review’ found in the Herschel family archives (belonging to John Shorland-Herschel).

## Author profile

**Emily Winterburn** is currently a visiting fellow at the University of Leeds, and was until recently curator at the Leeds University Museum for the History of Science, Technology and Medicine. She has a degree in physics, and now specializes in history of science research and curating. Between 1998 and 2007, she was curator of Astronomy at the Royal Observatory, Greenwich where she looked after a large collection relating to the Herschel family. Her interest in this collection gradually developed into a research project for which she was awarded her PhD in 2011. She is now turning this work into a book and has published articles from it in *Notes and Records of the Royal Society*, including one on Caroline Herschel, which complements this paper nicely. Besides the Herschels and history of astronomy, Emily's research interests also include the study of historic scientific instruments and the history of women's scientific education.
